# μ-Bis(diphenyl­arsino)methane-κ^2^ *As*:*As*′-bis­[chloridogold(I)]

**DOI:** 10.1107/S1600536809004802

**Published:** 2009-02-18

**Authors:** Uwe Monkowius, Manfred Zabel, Hartmut Yersin

**Affiliations:** a Institut für Anorganische Chemie, Johannes Kepler Universität Linz, Altenbergerstrasse 69, 4040 Linz, Austria; b Röntgenstrukturanalyse, Zentrale Analytik, Universität Regensburg, Universitätsstrasse 31, 93053 Regensburg, Germany; c Institut für Physikalische Chemie, Universität Regensburg, Universitätsstrasse 31, 93053 Regensburg, Germany

## Abstract

The title structure, [Au_2_Cl_2_(C_25_H_22_As_2_)], consists of discrete mol­ecules disposed about a crystallographic twofold axis. The Au atom exhibits a nearly linear coordination by As and Cl atoms. Au⋯Au inter­actions [3.4285Å(4) Å] and a weak intermolecular C—H⋯Cl hydrogen bond are present.

## Related literature

For related structures, see: Healy (2003 ▶); Schmidbaur *et al.* (1977*a* ▶,*b* ▶). For the synthesis of related complexes, see: Monkowius *et al.* (2003*a* ▶,*b* ▶).
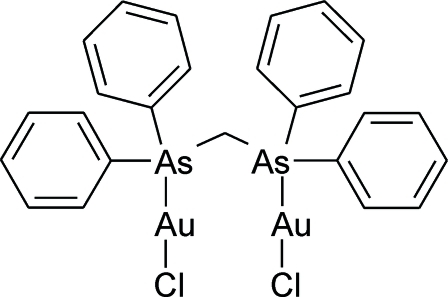


## Experimental

### 

#### Crystal data

[Au_2_Cl_2_(C_25_H_22_As_2_)]
*M* *_r_* = 937.11
Monoclinic, 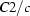

*a* = 22.7171 (18) Å
*b* = 7.3151 (6) Å
*c* = 18.2047 (15) Å
β = 120.342 (8)°
*V* = 2610.8 (4) Å^3^
*Z* = 4
Mo *K*α radiation
μ = 13.96 mm^−1^
*T* = 173 K
0.24 × 0.20 × 0.18 mm

#### Data collection

Stoe IPDS diffractometer
Absorption correction: analytical [from crystal shape; **X-SHAPE** and **X-RED** in **IPDS Software** (Stoe & Cie, 1998 ▶)] *T*_min_ = 0.051, *T*_max_ = 0.083
12353 measured reflections
2790 independent reflections
2431 reflections with *I* > 2σ(*I*)
*R*_int_ = 0.062

#### Refinement

*R*[*F* ^2^ > 2σ(*F* ^2^)] = 0.023
*wR*(*F* ^2^) = 0.055
*S* = 0.96
2790 reflections
142 parameters
H-atom parameters constrained
Δρ_max_ = 1.65 e Å^−3^
Δρ_min_ = −0.70 e Å^−3^

### 

Data collection: *IPDS Software* (Stoe & Cie, 1998 ▶); cell refinement: *IPDS Software*; data reduction: *IPDS Software*; program(s) used to solve structure: *SIR97* (Altomare *et al.*, 1999 ▶); program(s) used to refine structure: *SHELXL97* (Sheldrick, 2008 ▶); molecular graphics: *PLATON* (Spek, 2009 ▶); software used to prepare material for publication: *PLATON*.

## Supplementary Material

Crystal structure: contains datablocks global, I. DOI: 10.1107/S1600536809004802/nc2133sup1.cif

Structure factors: contains datablocks I. DOI: 10.1107/S1600536809004802/nc2133Isup2.hkl

Additional supplementary materials:  crystallographic information; 3D view; checkCIF report

## Figures and Tables

**Table:** 

Au1—As1	2.3426 (5)
Au1—Cl1	2.2887 (16)

**Table:** 

As1—Au1—Cl1	174.82 (4)

Symmetry code: (i) 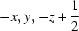.

**Table 2:** Hydrogen-bond geometry (Å, °)

*D*—H⋯*A*	*D*—H	H⋯*A*	*D*⋯*A*	*D*—H⋯*A*
C1—H1*A*⋯Cl1^ii^	0.99	2.70	3.658 (4)	163

Symmetry code: (ii) 
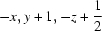
.

**Fig. 1.**
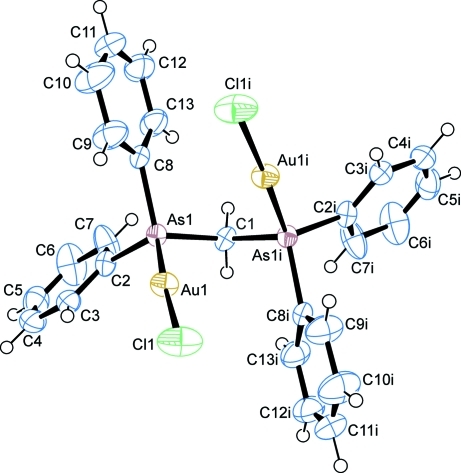
View of the title compound with the atom numbering scheme (symmetry code: (i) -x, y, -z + 1/2). Displacement ellipsoids for non-H atoms are drawn at the 50% probability level.

**Fig. 2.**
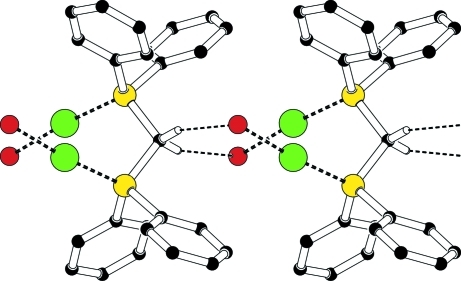
Crystal structure of the title compound depicting the intermolecular hydrogen bonds between H1A and Cl1ii (symmetry code: (ii) -x, y + 1, -z + 1/2). The H atoms not involved in hydrogen bonding have been omitted.

## supplementary crystallographic information

### Comment

The title compound was prepared from dpam [dpam = bis(diphenylarsino)methane] and (tht)AuCl (tht = tetrahydrothiophene) in methylene chloride in nearly quantitative yields. It is isomorphous to the crystal structure of the phosphorus congener [dppm(AuCl)_2_], which was determined by Schmidbaur *et al.* (1977*b*) [a = 22.31 (1) Å, b = 7.215 (7) Å, c = 18.12 (1) Å and β = 120.43 (8)°]. The structure consists of discrete molecules of [dpam(AuCl)_2_] disposed about a crystallographic twofold axis, which passes through the C1 atom. The Au atom is in a standard linear coordination [As—Au—Cl 174.82 (4)°] with As—Au and Au—Cl bond lengths of 2.3426 (5) and 2.289 (2) Å, respectively. The Au—As···As—Au torsion angle is 66.78 (2)°, yielding a staggered conformation of both Ph_2_AsAuCl moieties and an intramolecular Au···Au distance of 3.4285 (4) Å, indicative of attractive aurophilic interactions. The shortest intermolecular Au···Au distance is 5.863 Å. In its crystal, the complexes are linked to infinite chains *via* weak C—H···Cl intermolecular hydrogen bonds with C···Cl distance of 3.658 (4) Å and a C1—H1a···Cl1^ii^ angle of 163° (symmetry code: (ii) -*x*, *y* + 1, -*z* + 1/2).

For comparison, the geometrical data of the phosphorus compound are: Au—P 2.238 (5), Au—Cl 2.288 (7), Au···Au 3.351 (2) Å, P—Au—Cl 175 (2), Au—P···P—Au 67 (1)°. It should be noted, that a second polymorph of the phosphorus complex exists: Unlike the herein presented structure, there are no aurophilic bonds between the gold(I) atoms (Healy, 2003).

All attempts to prepare the 1:1 complex [(dpamAuCl)_2_] starting from the title compound analogous to the published synthesis of phosphorus complex [(dppmAuCl)_2_] (Schmidbaur *et al.*, 1977*a*) failed.

### Experimental

The title compound was prepared analogously to a previously published procedure (Monkowius *et al.*, 2003*a*,*b*): dpam (0.22 g, 0.47 mmol) and (tht)AuCl (0.30 g, 0.94 mmol, tht = tetrahydrothiophene) were stirred in methylene chloride (20 ml) at room temperature for 2 h. The product was precipitated with *n*-pentane and isolated by filtration. Recrystallization from methylene chloride/diethyl ether yields colourless crystals suitable for X-ray crystallography. Yield: 0.40 g (0.43 mmol, 91%); ^1^H NMR (300 MHz, CDCl_3_): 7.55–7.60 (m, Ph—H, 8 H), 7.37–7.51 (m, Ph—H, 12 H), 3.48 p.p.m. (s, CH_2_, 2 H); ^13^C NMR (75.5 MHz, CDCl_3_): 134.63, 132.93, 130.44, 129.23, 25.08 p.p.m.; MS (ESI): *m/z* (%) = 1605.3 [*L*_2_Au_3_Cl_2_]^+^ (5), 1373.3 [*L*_2_Au_3_Cl]^+^ (47), 1141.3 [*L*_2_Au]^+^ (76), 901.1 [M—Cl]^+^ (25), 669.2 [*L*Au]^+^ (100); EA (C_25_H_22_As_2_Au_2_Cl_2_) calc.: C 32.04, H 2.37, found: C 32.01, H 2.37.

### Refinement

The H atoms were positioned with idealized geometry and were refined isotropic using a riding model with C—H = 0.95 and 0.99 Å and *U*_iso_(H) = 1.2*U*_eq_(C).

### Crystal data

**Table:**

[Au_2_Cl_2_(C_25_H_22_As_2_)]	*F*(000) = 1720
*M**_r_* = 937.11	Cell parameters were determined by indexing 8000 reflections with I/sigma limit 6.0.
Monoclinic, *C*2/*c*	*D*_x_ = 2.384 Mg m^−^^3^
Hall symbol: -C 2yc	Mo *K*α radiation, λ = 0.71073 Å
*a* = 22.7171 (18) Å	Cell parameters from 8000 reflections
*b* = 7.3151 (6) Å	θ = 2.1–26.9°
*c* = 18.2047 (15) Å	µ = 13.96 mm^−^^1^
β = 120.342 (8)°	*T* = 173 K
*V* = 2610.8 (4) Å^3^	Prism, colourless
*Z* = 4	0.24 × 0.20 × 0.18 mm

### Data collection

**Table:**

Stoe IPDS diffractometer	2790 independent reflections
Radiation source: fine-focus sealed tube	2431 reflections with *I* > 2σ(*I*)
graphite	*R*_int_ = 0.062
rotation scans	θ_max_ = 26.9°, θ_min_ = 2.1°
Absorption correction: analytical [from crystal shape; *X-SHAPE* and *X-RED* in *IPDS Software* (Stoe & Cie, 1998)]	*h* = −28→28
*T*_min_ = 0.051, *T*_max_ = 0.083	*k* = −9→9
12353 measured reflections	*l* = −22→22

### Refinement

**Table:**

Refinement on *F*^2^	Secondary atom site location: difference Fourier map
Least-squares matrix: full	Hydrogen site location: inferred from neighbouring sites
*R*[*F*^2^ > 2σ(*F*^2^)] = 0.023	H-atom parameters constrained
*wR*(*F*^2^) = 0.055	*w* = 1/[σ^2^(*F*_o_^2^) + (0.0315*P*)^2^] where *P* = (*F*_o_^2^ + 2*F*_c_^2^)/3
*S* = 0.96	(Δ/σ)_max_ = 0.001
2790 reflections	Δρ_max_ = 1.65 e Å^−^^3^
142 parameters	Δρ_min_ = −0.70 e Å^−^^3^
0 restraints	Extinction correction: *SHELXL97* (Sheldrick, 2008), Fc^*^=kFc[1+0.001Fc^2^λ^3^/sin(2θ)]^-1/4^
Primary atom site location: structure-invariant direct methods	Extinction coefficient: 0.00085 (4)

### Special details

**Table:**

Experimental. Data were collected applying an imaging plate system (Stoe) with the following measurement parameters:Detector distance [mm] 65 Phi movement mode Oscillation Phi incr. [degrees] 1.2 Number of exposures 200 Irradiation / exposure [min] 2.00For a detailed description of the method see: Sheldrick, G. M., Paulus, E., Vertesy, L. & Hahn, F. (1995). Acta Cryst. B51, 89–98.
Geometry. Bond distances, angles *etc*. have been calculated using the rounded fractional coordinates. All su's are estimated from the variances of the (full) variance-covariance matrix. The cell e.s.d.'s are taken into account in the estimation of distances, angles and torsion angles
Refinement. Refinement of *F*^2^ against ALL reflections. The weighted *R*-factor *wR* and goodness of fit *S* are based on *F*^2^, conventional *R*-factors *R* are based on *F*, with *F* set to zero for negative *F*^2^. The threshold expression of *F*^2^ > σ(*F*^2^) is used only for calculating *R*-factors(gt) *etc*. and is not relevant to the choice of reflections for refinement. *R*-factors based on *F*^2^ are statistically about twice as large as those based on *F*, and *R*- factors based on ALL data will be even larger.

### Fractional atomic coordinates and isotropic or equivalent isotropic displacement parameters (Å^2^)

**Table:**

	*x*	*y*	*z*	*U*_iso_*/*U*_eq_	
Au1	0.00458 (1)	0.59917 (2)	0.15885 (1)	0.0364 (1)	
As1	−0.05684 (2)	0.86199 (5)	0.15224 (2)	0.0286 (1)	
Cl1	0.07339 (7)	0.35726 (17)	0.17281 (11)	0.0617 (5)	
C1	0.00000	1.0152 (8)	0.25000	0.0335 (16)	
C2	−0.08392 (19)	1.0208 (6)	0.0560 (3)	0.0345 (11)	
C3	−0.0783 (2)	0.9600 (7)	−0.0115 (3)	0.0430 (14)	
C4	−0.0981 (2)	1.0716 (9)	−0.0818 (3)	0.0553 (18)	
C5	−0.1228 (2)	1.2445 (8)	−0.0834 (3)	0.0583 (17)	
C6	−0.1289 (3)	1.3050 (8)	−0.0165 (4)	0.074 (2)	
C7	−0.1091 (3)	1.1941 (7)	0.0543 (3)	0.0640 (19)	
C8	−0.13949 (18)	0.8231 (6)	0.1565 (2)	0.0330 (10)	
C9	−0.1733 (3)	0.6586 (8)	0.1285 (4)	0.0591 (19)	
C10	−0.2351 (3)	0.6299 (9)	0.1269 (5)	0.078 (2)	
C11	−0.2608 (2)	0.7632 (10)	0.1543 (4)	0.0641 (18)	
C12	−0.2273 (3)	0.9248 (9)	0.1831 (4)	0.064 (2)	
C13	−0.1665 (2)	0.9580 (8)	0.1839 (3)	0.0515 (16)	
H1A	−0.02910	1.09450	0.26260	0.0400*	
H3A	−0.06070	0.84140	−0.01000	0.0520*	
H4A	−0.09480	1.02880	−0.12880	0.0660*	
H5A	−0.13560	1.32180	−0.13100	0.0700*	
H6A	−0.14670	1.42340	−0.01840	0.0890*	
H7A	−0.11280	1.23700	0.10110	0.0770*	
H9A	−0.15470	0.56450	0.11040	0.0720*	
H10A	−0.25880	0.51740	0.10660	0.0930*	
H11A	−0.30250	0.74360	0.15340	0.0760*	
H12A	−0.24560	1.01640	0.20300	0.0770*	
H13A	−0.14390	1.07230	0.20310	0.0620*	

### Atomic displacement parameters (Å^2^)

**Table:**

	*U*^11^	*U*^22^	*U*^33^	*U*^12^	*U*^13^	*U*^23^
Au1	0.0406 (1)	0.0289 (1)	0.0443 (1)	0.0048 (1)	0.0248 (1)	0.0023 (1)
As1	0.0285 (2)	0.0273 (2)	0.0273 (2)	0.0012 (1)	0.0121 (1)	−0.0007 (2)
Cl1	0.0723 (8)	0.0355 (6)	0.1015 (11)	0.0191 (6)	0.0618 (8)	0.0183 (7)
C1	0.026 (2)	0.032 (3)	0.034 (3)	0.0000	0.009 (2)	0.0000
C2	0.0340 (18)	0.032 (2)	0.0292 (19)	−0.0015 (16)	0.0098 (15)	0.0020 (16)
C3	0.0330 (19)	0.057 (3)	0.037 (2)	0.0046 (19)	0.0163 (17)	0.004 (2)
C4	0.041 (2)	0.087 (4)	0.041 (3)	0.004 (2)	0.023 (2)	0.014 (3)
C5	0.054 (3)	0.065 (3)	0.044 (3)	−0.007 (3)	0.016 (2)	0.017 (3)
C6	0.107 (5)	0.036 (3)	0.058 (3)	0.010 (3)	0.026 (3)	0.011 (3)
C7	0.106 (4)	0.038 (3)	0.038 (3)	0.010 (3)	0.029 (3)	0.001 (2)
C8	0.0248 (16)	0.041 (2)	0.0267 (18)	−0.0002 (16)	0.0082 (14)	−0.0010 (17)
C9	0.051 (3)	0.049 (3)	0.084 (4)	−0.013 (2)	0.039 (3)	−0.021 (3)
C10	0.058 (3)	0.071 (4)	0.108 (5)	−0.028 (3)	0.045 (4)	−0.019 (4)
C11	0.035 (2)	0.092 (4)	0.065 (3)	−0.008 (3)	0.025 (2)	0.001 (3)
C12	0.043 (3)	0.087 (4)	0.065 (4)	0.006 (3)	0.029 (2)	−0.015 (3)
C13	0.040 (2)	0.054 (3)	0.057 (3)	−0.006 (2)	0.022 (2)	−0.017 (2)

### Geometric parameters (Å, °)

**Table:**

Au1—As1	2.3426 (5)	C11—C12	1.360 (10)
Au1—Cl1	2.2887 (16)	C12—C13	1.395 (9)
As1—C1	1.941 (3)	C1—H1A	0.9900
As1—C2	1.926 (5)	C1—H1A^i^	0.9900
As1—C8	1.939 (5)	C3—H3A	0.9500
C2—C3	1.372 (7)	C4—H4A	0.9500
C2—C7	1.385 (7)	C5—H5A	0.9500
C3—C4	1.388 (7)	C6—H6A	0.9500
C4—C5	1.378 (9)	C7—H7A	0.9500
C5—C6	1.367 (8)	C9—H9A	0.9500
C6—C7	1.391 (8)	C10—H10A	0.9500
C8—C9	1.379 (8)	C11—H11A	0.9500
C8—C13	1.381 (7)	C12—H12A	0.9500
C9—C10	1.405 (11)	C13—H13A	0.9500
C10—C11	1.355 (10)		
			
Au1···C6^ii^	3.773 (6)	C1···H13A^i^	2.9600
Au1···C7^ii^	3.756 (6)	C1···H13A	2.9600
Au1···C4^iii^	3.910 (6)	C1···H7A	3.0900
Au1···C5^iii^	3.759 (5)	C3···H11A^viii^	3.0300
Au1···C8^i^	3.601 (4)	C4···H11A^viii^	3.0200
Au1···C9^i^	3.857 (7)	C13···H1A	2.8800
Au1···Au1^i^	3.4286 (4)	C13···H4A^ix^	2.9500
Au1···H7A^ii^	3.5200	H1A···C13	2.8800
Au1···H9A	3.2700	H1A···H13A	2.2700
Au1···H3A	3.1900	H1A···Cl1^x^	2.7000
Au1···H6A^ii^	3.5600	H3A···Au1	3.1900
Au1···H4A^iii^	3.6100	H4A···Au1^iii^	3.6100
Au1···H5A^iii^	3.3100	H4A···Cl1^v^	3.0400
Cl1···H1A^iv^	2.7000	H4A···C13^xi^	2.9500
Cl1···H13A^iv^	2.8900	H5A···Au1^iii^	3.3100
Cl1···H4A^v^	3.0400	H5A···Cl1^iii^	3.0300
Cl1···H5A^iii^	3.0300	H6A···Au1^vii^	3.5600
Cl1···H11A^vi^	3.1300	H7A···Au1^vii^	3.5200
C3···C3^iii^	3.416 (8)	H7A···C1	3.0900
C3···C4^iii^	3.481 (7)	H7A···H13A	2.5900
C4···Au1^iii^	3.910 (6)	H9A···Au1	3.2700
C4···C3^iii^	3.481 (7)	H11A···Cl1^xii^	3.1300
C5···Au1^iii^	3.759 (5)	H11A···C3^viii^	3.0300
C6···Au1^vii^	3.773 (6)	H11A···C4^viii^	3.0200
C7···Au1^vii^	3.756 (6)	H13A···C1	2.9600
C8···Au1^i^	3.601 (4)	H13A···H1A	2.2700
C9···Au1^i^	3.857 (7)	H13A···H7A	2.5900
C1···H7A^i^	3.0900	H13A···Cl1^x^	2.8900
			
As1—Au1—Cl1	174.82 (4)	As1—C1—H1A^i^	110.00
Au1—As1—C1	108.88 (12)	As1^i^—C1—H1A	110.00
Au1—As1—C2	116.75 (14)	H1A—C1—H1A^i^	108.00
Au1—As1—C8	116.18 (13)	As1^i^—C1—H1A^i^	110.00
C1—As1—C2	104.16 (19)	C2—C3—H3A	120.00
C1—As1—C8	104.90 (11)	C4—C3—H3A	120.00
C2—As1—C8	104.72 (19)	C3—C4—H4A	120.00
As1—C1—As1^i^	109.5 (3)	C5—C4—H4A	120.00
As1—C2—C3	119.2 (3)	C4—C5—H5A	120.00
As1—C2—C7	120.7 (4)	C6—C5—H5A	120.00
C3—C2—C7	120.1 (4)	C5—C6—H6A	120.00
C2—C3—C4	120.1 (5)	C7—C6—H6A	120.00
C3—C4—C5	119.9 (5)	C2—C7—H7A	120.00
C4—C5—C6	120.1 (5)	C6—C7—H7A	120.00
C5—C6—C7	120.4 (6)	C8—C9—H9A	120.00
C2—C7—C6	119.4 (5)	C10—C9—H9A	120.00
As1—C8—C9	118.9 (4)	C9—C10—H10A	120.00
As1—C8—C13	121.7 (4)	C11—C10—H10A	120.00
C9—C8—C13	119.4 (5)	C10—C11—H11A	120.00
C8—C9—C10	120.1 (6)	C12—C11—H11A	120.00
C9—C10—C11	119.8 (6)	C11—C12—H12A	119.00
C10—C11—C12	120.4 (6)	C13—C12—H12A	120.00
C11—C12—C13	120.9 (6)	C8—C13—H13A	120.00
C8—C13—C12	119.3 (5)	C12—C13—H13A	120.00
As1—C1—H1A	110.00		
			
Au1—As1—C1—As1^i^	−34.87 (4)	C3—C2—C7—C6	0.4 (9)
C2—As1—C1—As1^i^	−160.10 (16)	C7—C2—C3—C4	−0.3 (8)
C8—As1—C1—As1^i^	90.12 (13)	As1—C2—C7—C6	−179.4 (5)
Au1—As1—C2—C3	12.2 (5)	C2—C3—C4—C5	0.7 (8)
C1—As1—C2—C3	132.3 (4)	C3—C4—C5—C6	−1.1 (8)
C8—As1—C2—C3	−117.9 (4)	C4—C5—C6—C7	1.2 (10)
Au1—As1—C2—C7	−168.1 (4)	C5—C6—C7—C2	−0.8 (10)
C1—As1—C2—C7	−48.1 (5)	As1—C8—C9—C10	−176.9 (5)
C8—As1—C2—C7	61.9 (5)	C13—C8—C9—C10	0.8 (8)
C2—As1—C8—C9	104.0 (4)	As1—C8—C13—C12	177.9 (4)
Au1—As1—C8—C9	−26.4 (4)	C9—C8—C13—C12	0.3 (7)
C1—As1—C8—C9	−146.6 (4)	C8—C9—C10—C11	−1.1 (10)
C2—As1—C8—C13	−73.6 (4)	C9—C10—C11—C12	0.2 (11)
Au1—As1—C8—C13	156.0 (3)	C10—C11—C12—C13	1.0 (10)
C1—As1—C8—C13	35.7 (4)	C11—C12—C13—C8	−1.2 (8)
As1—C2—C3—C4	179.4 (4)		

Symmetry codes: (i) −*x*, *y*, −*z*+1/2; (ii) *x*, *y*−1, *z*; (iii) −*x*, −*y*+2, −*z*; (iv) −*x*, *y*−1, −*z*+1/2; (v) −*x*, −*y*+1, −*z*; (vi) *x*+1/2, *y*−1/2, *z*; (vii) *x*, *y*+1, *z*; (viii) −*x*−1/2, −*y*+3/2, −*z*; (ix) *x*, −*y*+2, *z*+1/2; (x) −*x*, *y*+1, −*z*+1/2; (xi) *x*, −*y*+2, *z*−1/2; (xii) *x*−1/2, *y*+1/2, *z*.

### Hydrogen-bond geometry (Å, °)

**Table:**

*D*—H···*A*	*D*—H	H···*A*	*D*···*A*	*D*—H···*A*
C1—H1A···Cl1^x^	0.99	2.70	3.658 (4)	163

Symmetry codes: (x) −*x*, *y*+1, −*z*+1/2.
